# Case Report: Treatment of trachyonychia in children with crisaborole: a retrospective case series

**DOI:** 10.3389/fped.2026.1865351

**Published:** 2026-07-15

**Authors:** Xue Wang, Keying Li, Yuanxiang Liu, Rui He, Yujuan Sun, Zigang Xu, Bin Zhang

**Affiliations:** 1 Department of Dermatology, Beijing Children’s Hospital, Capital Medical University (National Center for Children’s Health, China), Beijing, China; 2 Key Laboratory of Major Diseases in Children, Ministry of Education, Beijing, China

**Keywords:** children, crisaborole, effective, safe, trachyonychia

## Abstract

**Background:**

Trachyonychia is a chronic nail disorder affecting children aged 3–12 years, characterized by nail plate roughness and longitudinal ridging. It exhibits pathological features similar to atopic dermatitis, including spongiotic edema and inflammatory cell infiltration of the nail epithelium. Most pediatric cases are idiopathic, and high-quality therapeutic evidence remains limited. Crisaborole, a novel nonsteroidal PDE4 inhibitor with potent anti-inflammatory activity, may represent a promising therapeutic strategy.

**Methods:**

This retrospective study was conducted at the Department of Dermatology, Beijing Children's Hospital between April 2021 and September 2022. Thirteen children diagnosed with trachyonychia were enrolled, with onychomycosis excluded by KOH examination. All patients received topical crisaborole twice daily on the nail plates and nail folds with nightly occlusion. Outcomes were assessed at 6 and 12 weeks using PGA and FDLQI. Statistical analysis was performed using the Wilcoxon signed-rank test in SPSS 24.0, with *P* < 0.05 considered statistically significant.

**Results:**

Thirteen children (9 males, 4 females; mean age 7.7 ± 2.2 years) with 95 affected nails were analyzed. At week 6, 82.1% of nails showed significant clinical improvement, including 43.2% complete remission and 38.9% partial remission. PGA and FDLQI scores were significantly reduced (*P* < 0.05). At week 12, 48.5% of nails maintained complete remission. No severe adverse events were reported.

**Conclusion:**

Crisaborole is effective, safe, and well-tolerated for pediatric trachyonychia with favorable compliance. Its small molecular weight facilitates nail matrix penetration and targets the shared inflammatory pathogenesis of trachyonychia and atopic dermatitis. Crisaborole therefore represents a promising and reliable therapeutic option for children with trachyonychia.

## Introduction

Trachyonychia is a chronic nail condition characterized by roughness and longitudinal ridging of the nail plates (1). It is more frequently observed in children aged from 3 to 12 years old and has a protracted course (2). Clinical diagnosis relies primarily on characteristic clinical features and onychoscopic findings, with nail matrix biopsy being reserved solely for severe, refractory cases or those with diagnostic uncertainty. Spongiosis and exocytosis of inflammatory cells into the nail epithelia are the most common features of trachyonychia, which is also a characteristic of atopic dermatitis (AD) (1). In children, trachyonychia is more commonly idiopathic, in a few cases, alopecia areata (AA), lichenoid planus and psoriasis can be the reasons of trachyonychia. Many of the pediatric patients and their families desire to improve appearance. The management of pediatric trachyonychia is currently geared primarily toward symptomatic relief and tailored to disease severity as well as concomitant disorders. Topical agents such as urea-containing ointments, topical corticosteroids and calcipotriol/betamethasone dipropionate ointment constitute the first-line therapy for the majority of cases, however, super-potent topical corticosteroids, despite their efficacy in alleviating inflammatory insults to the nail matrix, carry risks of local cutaneous adverse events (e.g., cutaneous atrophy, striae formation) with prolonged use—particularly concerning in children with delicate periungual skin (2, 3). Whereas systemic treatments and intramatrical injections are confined to severe, refractory cases with underlying comorbidities, systemic therapeutic modalities in the pediatric population are plagued by a high incidence of adverse events, and intramatrical injections are associated with poor treatment adherence in children due to the invasive nature of the procedure (2–4). Notably, a standardized therapeutic protocol for this condition is still not established, and there is limited data of treatment for pediatric trachyonychia. Crisaborole, a novel nonsteroidal small molecule drug targeted at PDE4 (phosphodiesterase-4), inhibits many inflammations applied for treatment in AD, which might be a promising strategy for trachyonychia.

## Methods

This case-series study was conducted from April 2021 to September 2022 at the Dermatology Department in Beijing Children's Hospital. All patients enrolled who presented with typical roughness and longitudinal ridging of the thin nail plates and underwent KOH examinations to rule out onychomycosis were diagnosed with trachyonychia. Crisaborole was applied topically over nail and nail folds twice daily, with plastic film occlusion for 4–6 h nightly. No additional emollients were applied throughout the treatment course. The first follow-up was 6 weeks later, and the second follow-up was 12 weeks later. The PGA (physician's global assessment, degree of roughness: stage 0, clear; stage I, mild; stage II, moderate; stage III, marked; stage IV, severe) (5) and the FDLQI (family dermatology life quality index) were estimated. Treatment response was categorized as follows: complete response (CR) was defined as complete resolution of trachyonychia lesions, and partial response (PR) was defined as improvement of ≥ 1 PGA stage. The level of significance assessed was *P* < 0.05. A Wilcoxon paired test was performed by SPSS 24.0 to evaluate the significance of differences in the outcome.

## Results

A total of 13 children were included: nine males and four females. The average age was 7.7 ± 2.2 years old, and the duration of the disease was 14.0 ± 13.1 months. The baseline characteristics of all enrolled patients are detailed in Table 1. 11 children were treatment-naïve for trachyonychia. Both Patient 4 and Patient 10 were previously treated with topical mometasone furoate; the former had no treatment response, and the latter exhibited no obvious improvement. No additional systemic or topical therapies were used before enrollment. Notably, a substantial proportion of patients presented with concurrent systemic or allergic conditions. Specifically, 30.77% (4/13) were complicated by atopic dermatitis, 2 (15.38%) cases by allergic diseases, and 1 by alopecia areata, highlighting a high prevalence of immune-related comorbidities in this pediatric cohort. Furthermore, family history analysis identified a positive familial tendency toward atopic disorders, with a family history of eczema and psoriasis documented in 1 patient, and a family history of alopecia areata in another patient. Additionally, environmental triggers were identified in 2 cases, including a history of dance/kickboxing-related trauma and a persistent nail-picking habit, suggesting potential contributions of both genetic predisposition and environmental factors to the pathogenesis of trachyonychia in children.

**Table 1:** Baseline characteristics of children with trachyonychia.

Cases	Age	Gender	Disease Course (month)	Type	Associated Diseases	Family History	Notes
1	4y5m	Male	12	Shinny			
2	6y9m	Male	60	Opaque	AD		
3	11y5m	Male	12	Shinny			
4	6y2m	Female	6	Opaque			Mometasone furoate for 2 weeks, with worsening
5	5y6m	Male	2	Opaque			
6	7y1m	Female	8	Opaque			Dance/kickboxing trauma history
7	6y9m	Female	3	Shinny	Allergic rhinitis		
8	10y9m	Male	17	Opaque			Nail picking habit
9	9y5m	Female	12	Opaque	AD		
10	6y10m	Male	2	Opaque	AD, allergic rhinitis and pharyngitis, attention deficit hyperactivity disorder (ADHD)	Eczema in parents, psoriasis in grandfather	Mometasone furoate: no significant improvement
11	8y	Male	24	Opaque			
12	10y11m	Male	12	Opaque	AD		
13	7y	Male	12	Opaque	AA	AA in uncle	

**Figure 1:**
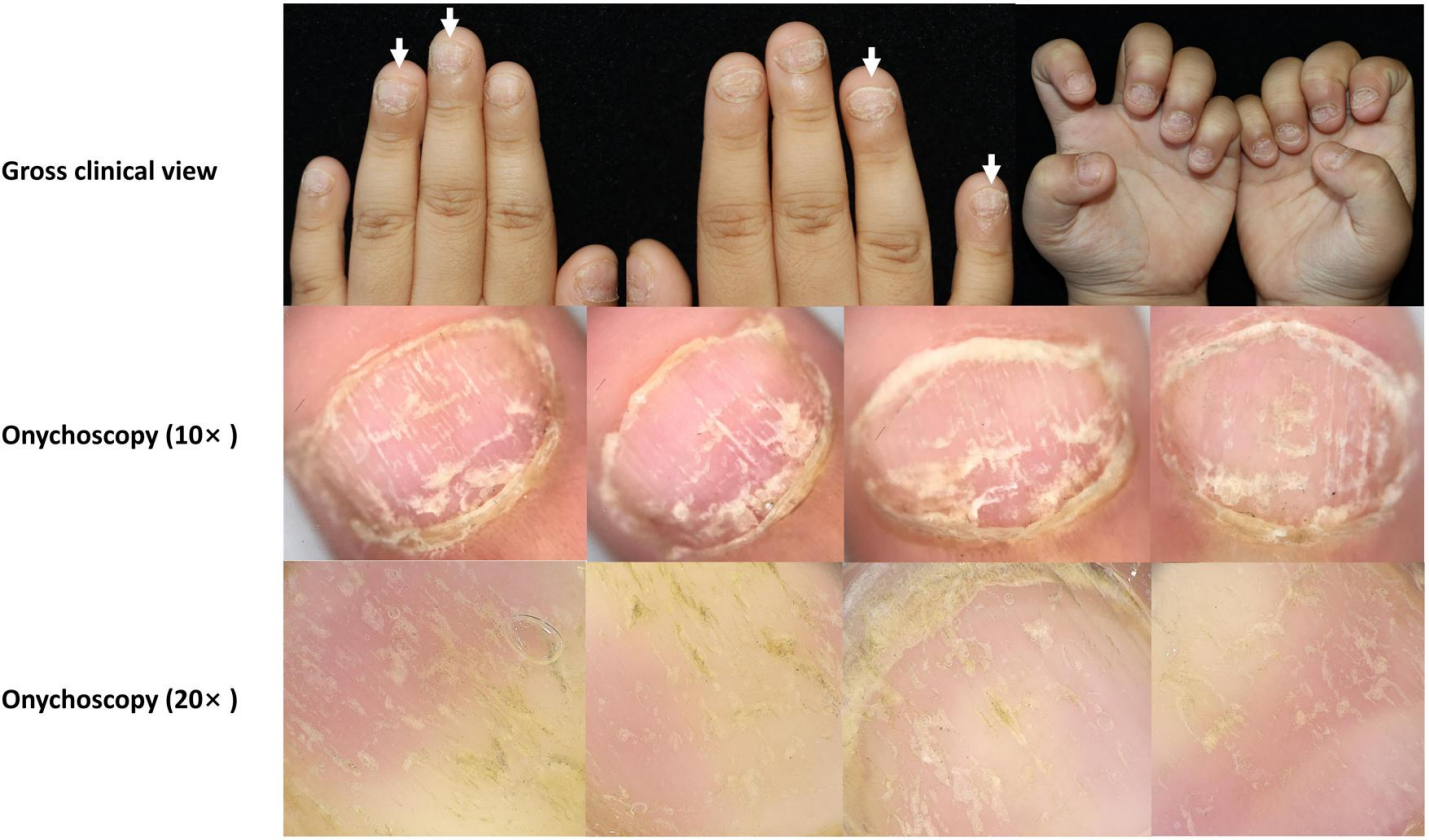
Pretreatment clinical and onychoscopic manifestations of pediatric trachyonychia. The top row shows the overall gross appearance of the patient's fingernails; white arrows denote the nails selected for dermoscopic observation. The middle row contains 10 ×  magnified onychoscopic images of these arrow-marked nails, presenting diffuse longitudinal ridging, a rough and dull nail plate surface, opacification, and nail plate thinning. The bottom row displays at 20 ×   magnification, distal splitting of the nail plate was additionally observed, along with the changes seen at 10 ×  magnification.

**Figure 2:**
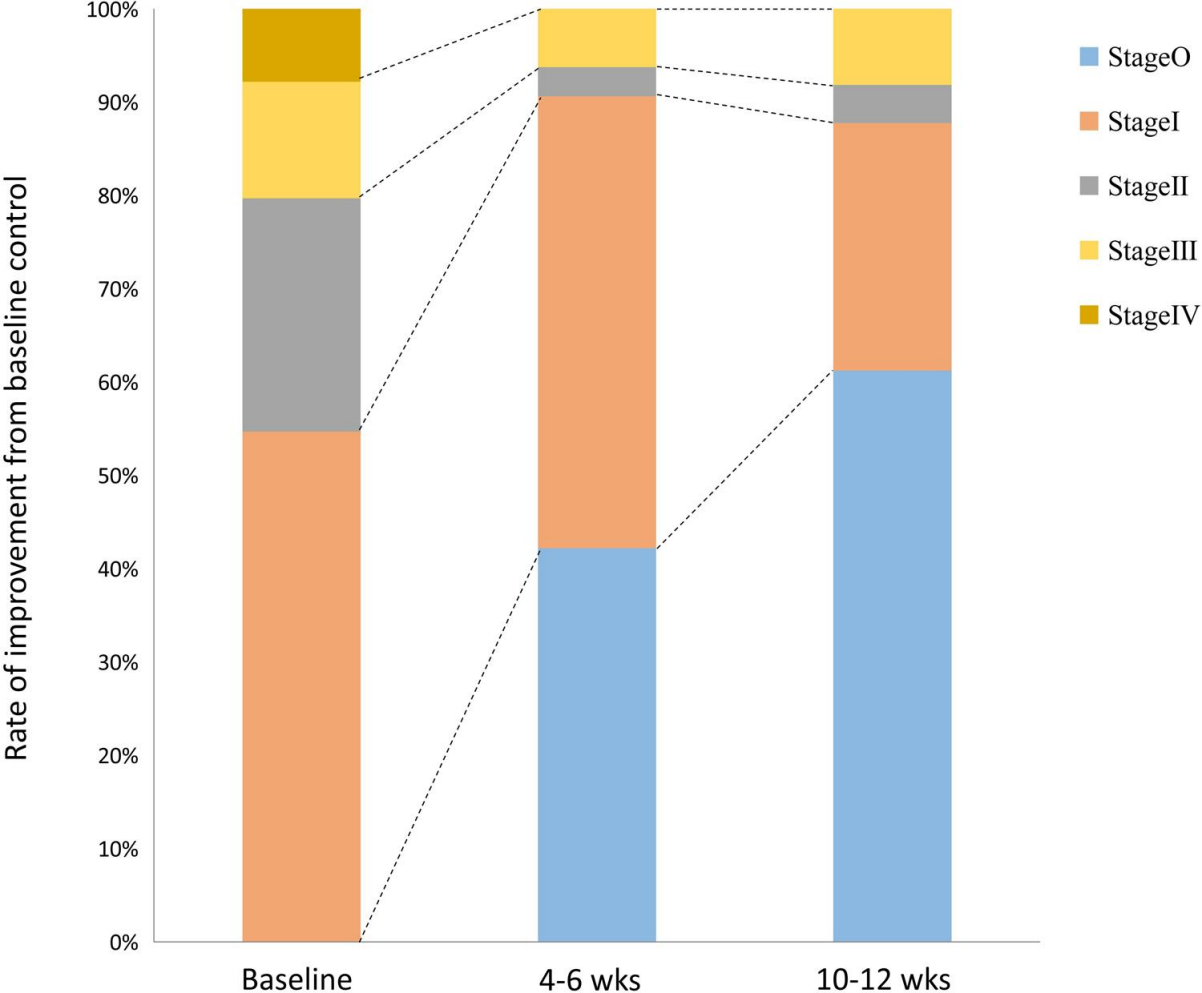
Clinical improvement of trachyonychia nails stratified by PGA stage over the course of topical crisaborole treatment. At baseline, 95 affected nails were classified into stage I (*n* = 44), stage II (*n* = 19), stage III (*n* = 15), and stage IV (*n* = 17). The stacked bars represent the distribution of nails across PGA stages at baseline, 4–6 weeks, and 10–12 weeks of treatment, with the *y*-axis indicating the rate of improvement from baseline. The dashed lines illustrate the cumulative shift toward lower PGA stages over time, reflecting progressive clinical improvement with therapy. PGA, Physician's Global Assessment.

**Figure 3:**
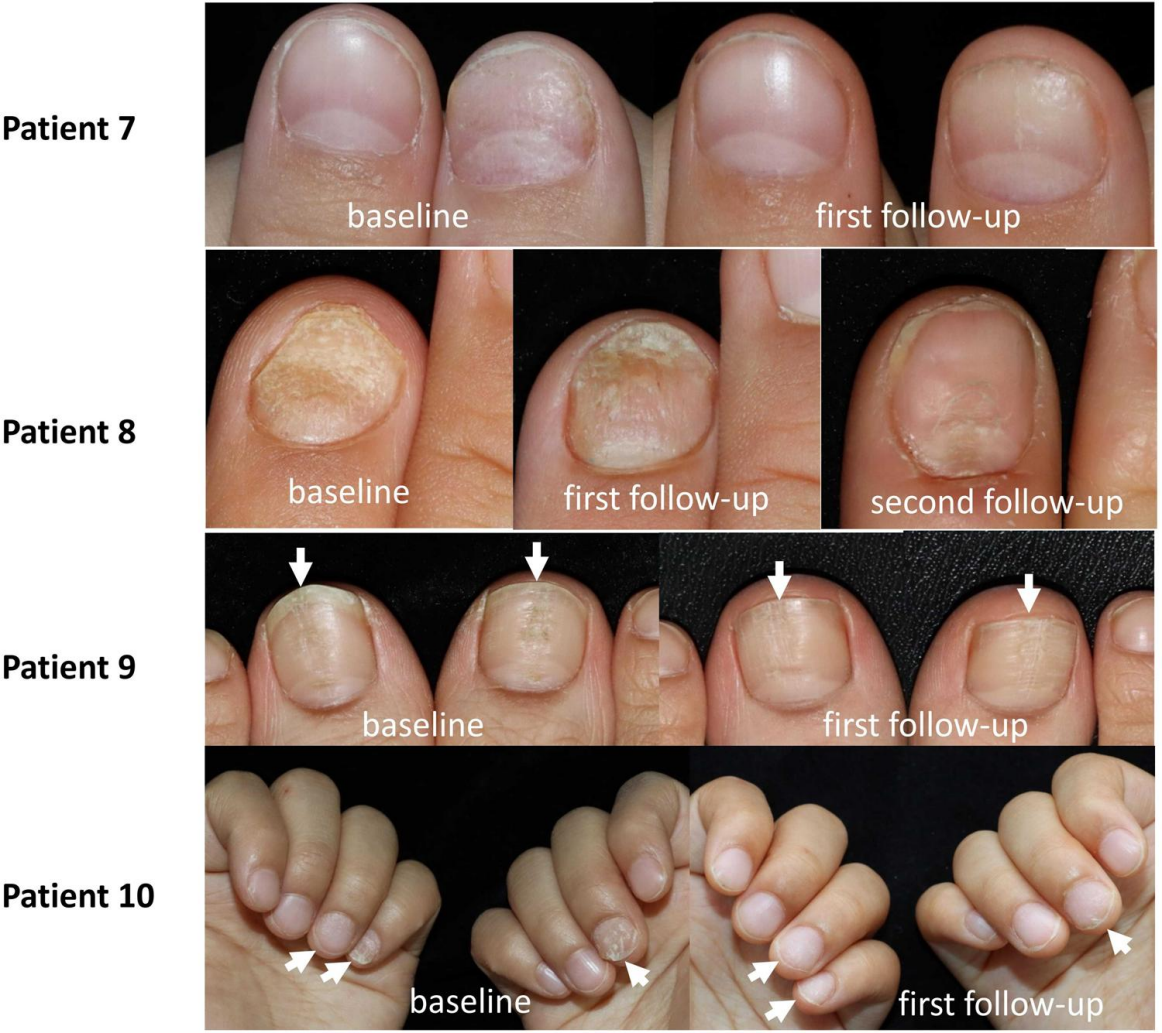
Representative clinical photographs of children with trachyonychia demonstrating improvement after treatment with topical crisaborole. Sequential images show baseline trachyonychia features (rough, pitted, and dystrophic nail plates) and corresponding improvements at first (4–6 weeks) and/or second (10–12 weeks) follow-up visits. White arrows highlight areas of clinical improvement in affected nails.

Overall, 95 nails affected by trachyonychia were evaluated at baseline (Figure 1), with 44 classified as stage I, 19 as stage II, 15 as stage III, and 17 as stage IV (Figure 2); the mean PGA score was 2.1 ± 1.1, and the median FDLQI score was 7. After 6 weeks of topical crisaborole therapy, 82.1% (78/95) of nails demonstrated significant clinical improvement, including 43.2% (41/95) achieving CR and 38.9% (37/95) achieving PR. The mean PGA score decreased significantly (*p* < 0.05), and the median FDLQI score reduced to 3, indicating a marked improvement in patients' quality of life. At the 12-week follow-up, the complete response rate reached 48.5% (47/95), with 18 nails prematurely withdrawn from follow-up due to early clinical improvement after the first assessment (Figure 3). No serious treatment-related adverse events were reported throughout the study period.

## Discussion

There is currently no evidence-based therapy available for pediatric trachyonychia, which is characterized by persistent inflammatory insult to the nail matrix and subsequent abnormal keratinization of the nail plate (6). We differentiated pediatric trachyonychia from a range of infectious and inflammatory nail disorders with similar presentations. First, onychomycosis was excluded: it generally manifests as nail discoloration, thickening, subungual hyperkeratosis and onycholysis, and tends to involve one or a few nails asymmetrically (7). KOH microscopic examination was negative for fungal hyphae and spores in all patients. We further excluded two major inflammatory nail diseases. Nail psoriasis features variable pits, salmon spots, splinter hemorrhages and severe subungual hyperkeratosis, and is usually associated with cutaneous psoriasis (8, 9), with no such findings in our subjects. Nail lichen planus is marked by nail thinning, longitudinal fissures and pathognomonic pterygium unguis, and may cause permanent nail damage (10, 11), which were not present in our cohort. We also ruled out nail lesions secondary to alopecia areata and AD. Our patients only had mild alopecia, inconsistent with typical alopecia-related nail changes (12). Some children had AD history, yet no characteristic periungual inflammation, eczematous lesions or nail picking was noted, excluding AD-induced nail dystrophy (8).

Our findings showed that the PDE4 inhibitor was effective and generally adhered to in the treatment of pediatric trachyonychia. Patients who received topical crisaborole experienced improvement and resolution with minimal side effects. With a molecular weight of 251 Da and unique boron-based chemical structure, crisaborole might have the ability to penetrate effectively through the proximal nail fold to the nail matrix—the primary pathological site of trachyonychia—thus exerting targeted local anti-inflammatory effects while eliciting minimal systemic exposure, as the drug is rapidly metabolized into inactive metabolites *in vivo* with no subsequent PDE4 inhibitory or cytokine-modulating activity (13). Furthermore, trachyonychia is histopathologically typified by spongiosis and inflammatory cell exocytosis into the nail epithelium (6, 12), features that overlap with atopic dermatitis (AD) (1). Crisaborole has been well-established as an effective and targeted topical therapy for the treatment of AD in both pediatric and adult populations (14). We therefore postulate that crisaborole may exert its therapeutic efficacy in pediatric trachyonychia via a similar mechanism, which suppresses proinflammatory signaling pathways and mitigates the inflammatory damage to the nail matrix that underlies trachyonychia (13, 14). The petrolatum-based emollient vehicle of crisaborole ointment not only exerts a direct reparative effect on the impaired periungual skin barrier, but also further enhances the improvement of classic clinical manifestations of the disorder, including nail roughness, pitting, longitudinal ridging and koilonychia (6). This is consistent with the well-documented vehicle effect in atopic dermatitis (AD) clinical research (13, 14). As evidenced by the phase III clinical trials of crisaborole for AD, the petrolatum vehicle alone can yield significant clinical improvement by restoring cutaneous barrier integrity, reducing transepidermal water loss, and alleviating mild local inflammation in lesional skin (14). These pharmacodynamic properties of the vehicle are equally applicable to the periungual microenvironment in trachyonychia, the highly lipophilic characteristic of the petrolatum vehicle enables prolonged adherence to the proximal nail fold and periungual skin, this prolonged contact enhances hydration of the entire nail unit and exerts a direct symptomatic ameliorative effect on the physical dystrophic changes of the nail plate. Collectively, the clinical improvements observed with topical crisaborole in pediatric trachyonychia are likely the result of a synergistic action between the specific anti-inflammatory efficacy of the PDE4 inhibitor on nail matrix inflammation and the non-specific barrier-repairing, anti-inflammatory and symptomatic benefits of its petrolatum vehicle.

Crisaborole is a good option for the patient who desires improvement in a short time. On the one hand, spontaneous improvement cannot be reached in a short time. In a long-term follow-up of eleven patients with pediatric trachyonychia, the condition improved on average after about 66 months (15). On the other hand, another research shown that without therapy, the patients with childhood onset are unlikely to recover (16).

Due to the limited sample size and period of follow-up in this retrospective study, coupled with the absence of a control group, we are unable to draw any further conclusions, and future prospective research is warranted.

## Conclusion

Trachyonychia is a common but therapeutically challenging nail disorder in children, with limited evidence-based treatment options available. In this retrospective case series, we described thirteen pediatric patients with trachyonychia treated with topical crisaborole, systematically summarizing their clinical presentation, treatment regimen, and therapeutic outcomes. Topical crisaborole demonstrated meaningful clinical efficacy in relatively short time. The treatment was well-tolerated, with no severe adverse events reported and favorable compliance. Although the small sample size and lack of a control group limit the generalizability of our observations, the rarity of targeted therapies for this condition makes detailed case-based data clinically informative. Crisaborole represents a promising therapeutic option that warrants validation in future prospective controlled studies.

## Data Availability

The original contributions presented in the study are included in the article, further inquiries can be directed to the corresponding author.
